# Supplementary material for: MOB rules: Antibiotic Exposure Reprograms Metabolism to Mobilize Bacillus subtilis in Competitive Interactions
Source: bioRxiv. 2024 Mar 20:2024.03.20.585991. Preprint. [Version 1] doi: 10.1101/2024.03.20.585991 (PMC10983992; doi:10.1101/2024.03.20.585991)
Supplement: Supplement 9 [file NIHPP2024.03.20.585991v1-supplement-9.pdf]

bioRxiv preprint doi: https://doi.org/10.1101/2024.03.20.585991; this version posted March 20, 2024. The copyright holder for this preprint (which was not certified by peer review) is the author/funder, who has granted bioRxiv a license to display the preprint in perpetuity. It is made available under aCC-BY 4.0 International license.

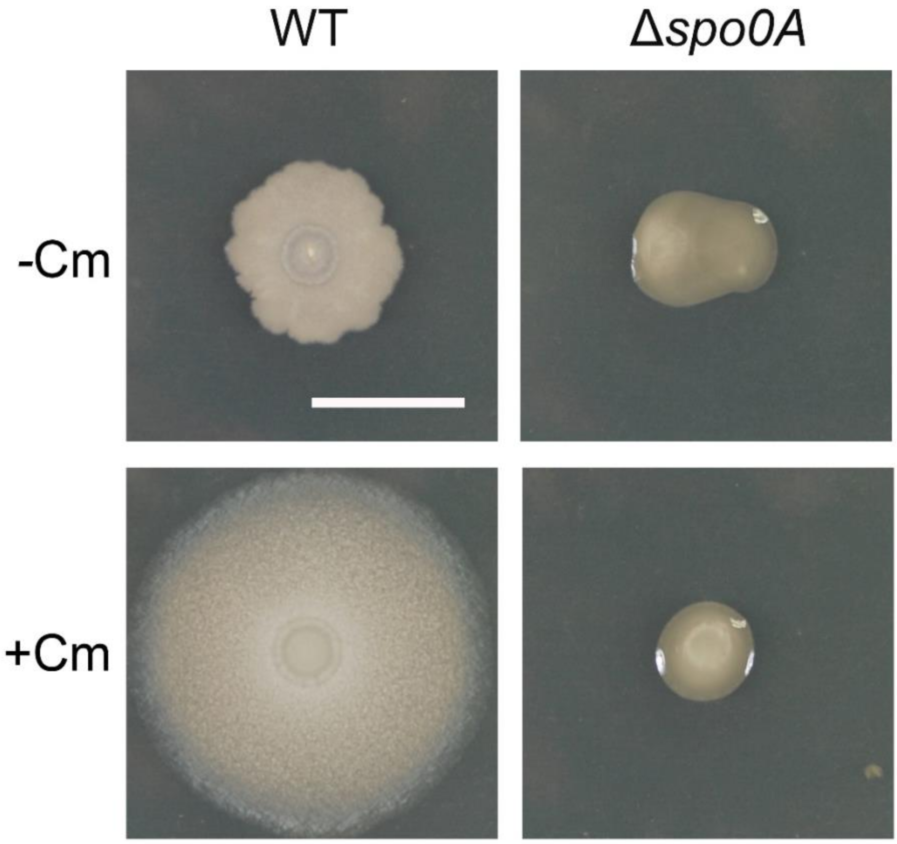

Figure S1. The colony of Δ*spo0A* strain was unable to expand in the presence of chloramphenicol.

Wild type (WT) and Δ*spo0A B. subtilis* NCIB3610 were spotted on the GYM7 plate in the absence (-Cm) and presence (+Cm) of chloramphenicol. Pictures were taken at 24 h. Bar, 1 cm.

bioRxiv preprint doi: https://doi.org/10.1101/2024.03.20.585991; this version posted March 20, 2024. The copyright holder for this preprint (which was not certified by peer review) is the author/funder, who has granted bioRxiv a license to display the preprint in perpetuity. It is made available under aCC-BY 4.0 International license.

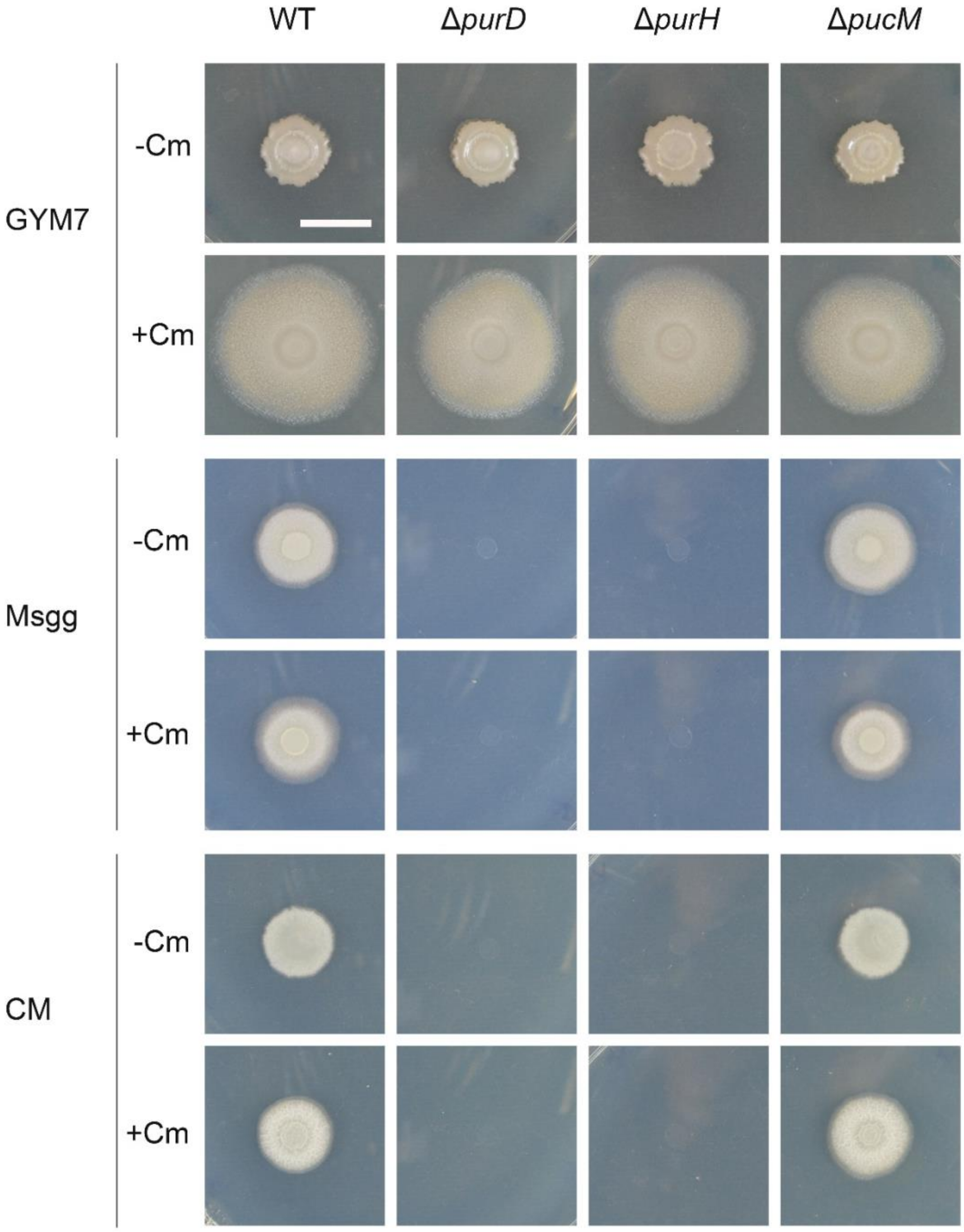

Figure S2. **Disruption of purine de novo biosynthesis pathway or catabolism pathway has different effects on *B. subtilis* growth under different conditions.** Wild type, Δ*purD*, Δ*purH*, and Δ*purM B. subtilis* NCIB3610 were spotted on the agar plate (1.5% agar) with GYM7 (top), Msgg (middle), and CM (bottom) media in the absence (-Cm) and presence (+Cm) of chloramphenicol. Pictures were taken at 24 h. Bar, 1 cm.

bioRxiv preprint doi: https://doi.org/10.1101/2024.03.20.585991; this version posted March 20, 2024. The copyright holder for this preprint (which was not certified by peer review) is the author/funder, who has granted bioRxiv a license to display the preprint in perpetuity. It is made available under aCC-BY 4.0 International license.

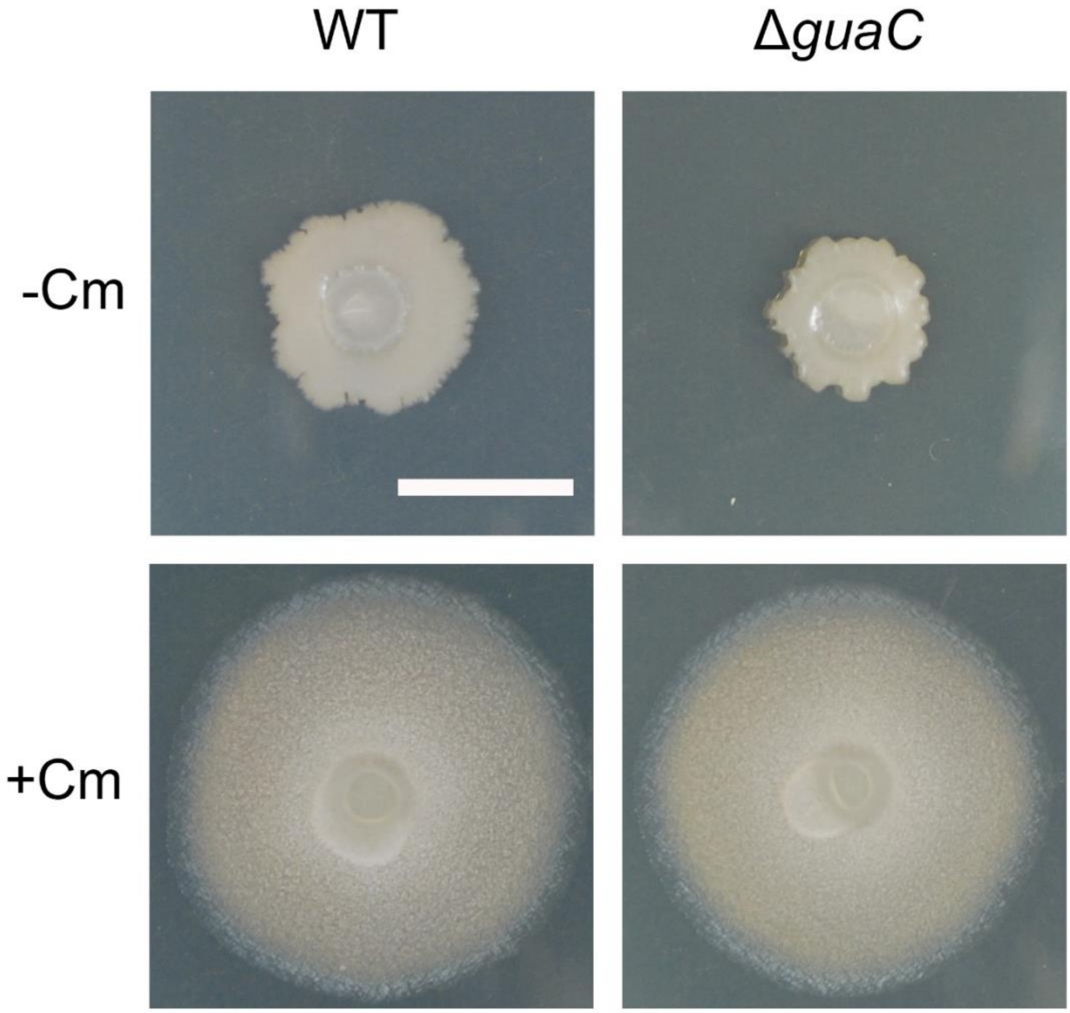

Figure S3. The colony of Δ*guaC* strain was able to expand in the presence of chloramphenicol.

Wild type (WT) and Δ*guaC B. subtilis* NCIB3610 were spotted on the GYM7 plate in the absence (-Cm) and presence (+Cm) of chloramphenicol. Pictures were taken at 24 h. Bar, 1 cm.

bioRxiv preprint doi: https://doi.org/10.1101/2024.03.20.585991; this version posted March 20, 2024. The copyright holder for this preprint (which was not certified by peer review) is the author/funder, who has granted bioRxiv a license to display the preprint in perpetuity. It is made available under aCC-BY 4.0 International license.

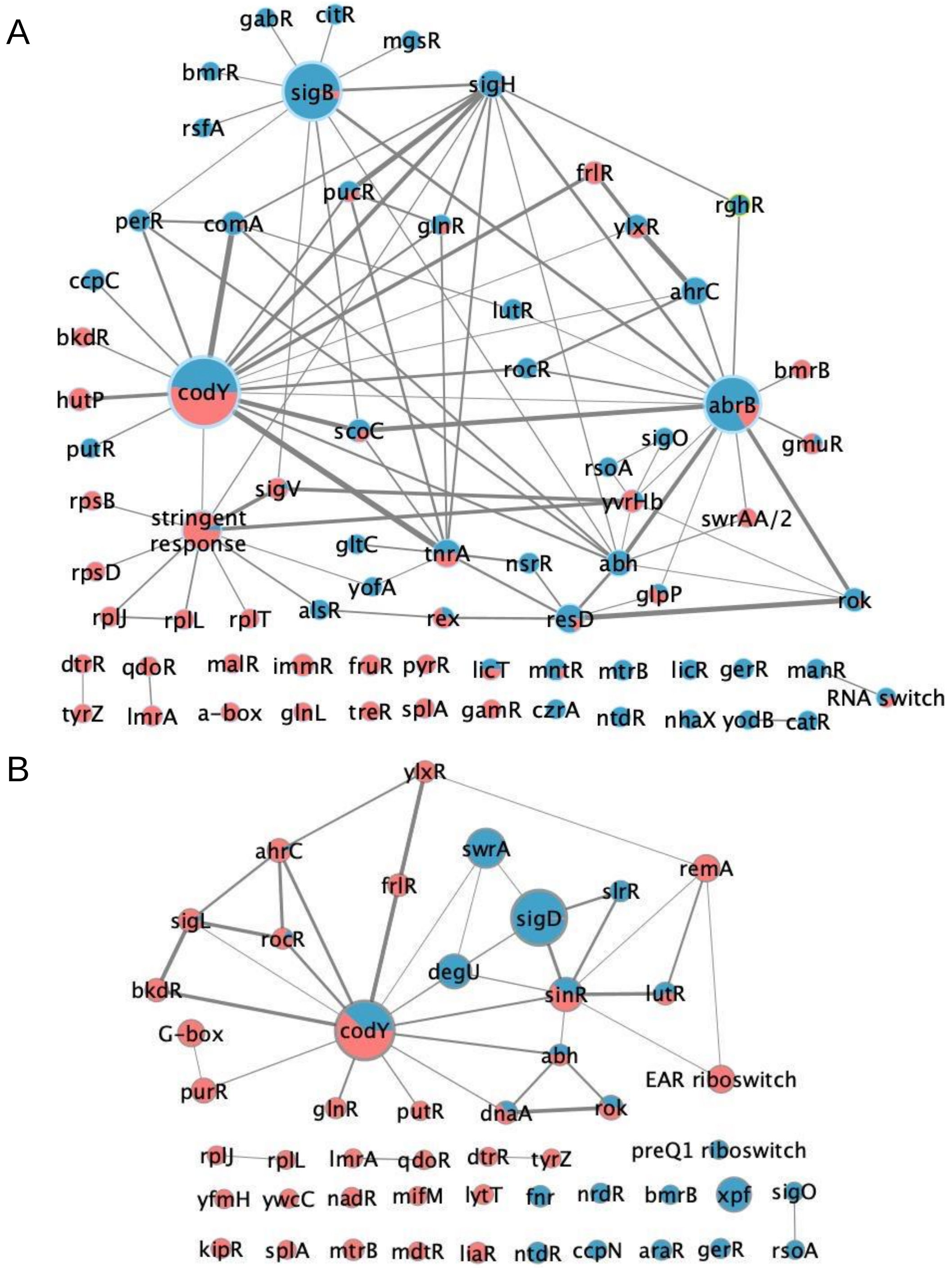

Figure S4. A network view Δ*codY* regulation in a constitutively mobile population. Cytoscape networks of regulators with ≥ 40% engagement of regulated genes at either: **A.** 6 h Δ*codY* gene expression ≥ 2-fold difference from wildtype controls with adjusted p-value < 0.05, or **B.** 24 h Δ*codY* gene expression ≥ 2-fold difference from untreated controls with adjusted p-value < 0.05. In both A and B, the size of each node represents weighted gene number in each regulon (weighted by percentage of differentially expressed genes in each regulon) and the edge width represents weighted overlapped gene number between two regulons (weighted by percentage of differentially expressed genes among overlapped genes). Blue: downregulation; Red: upregulation.

bioRxiv preprint doi: https://doi.org/10.1101/2024.03.20.585991; this version posted March 20, 2024. The copyright holder for this preprint (which was not certified by peer review) is the author/funder, who has granted bioRxiv a license to display the preprint in perpetuity. It is made available under aCC-BY 4.0 International license.

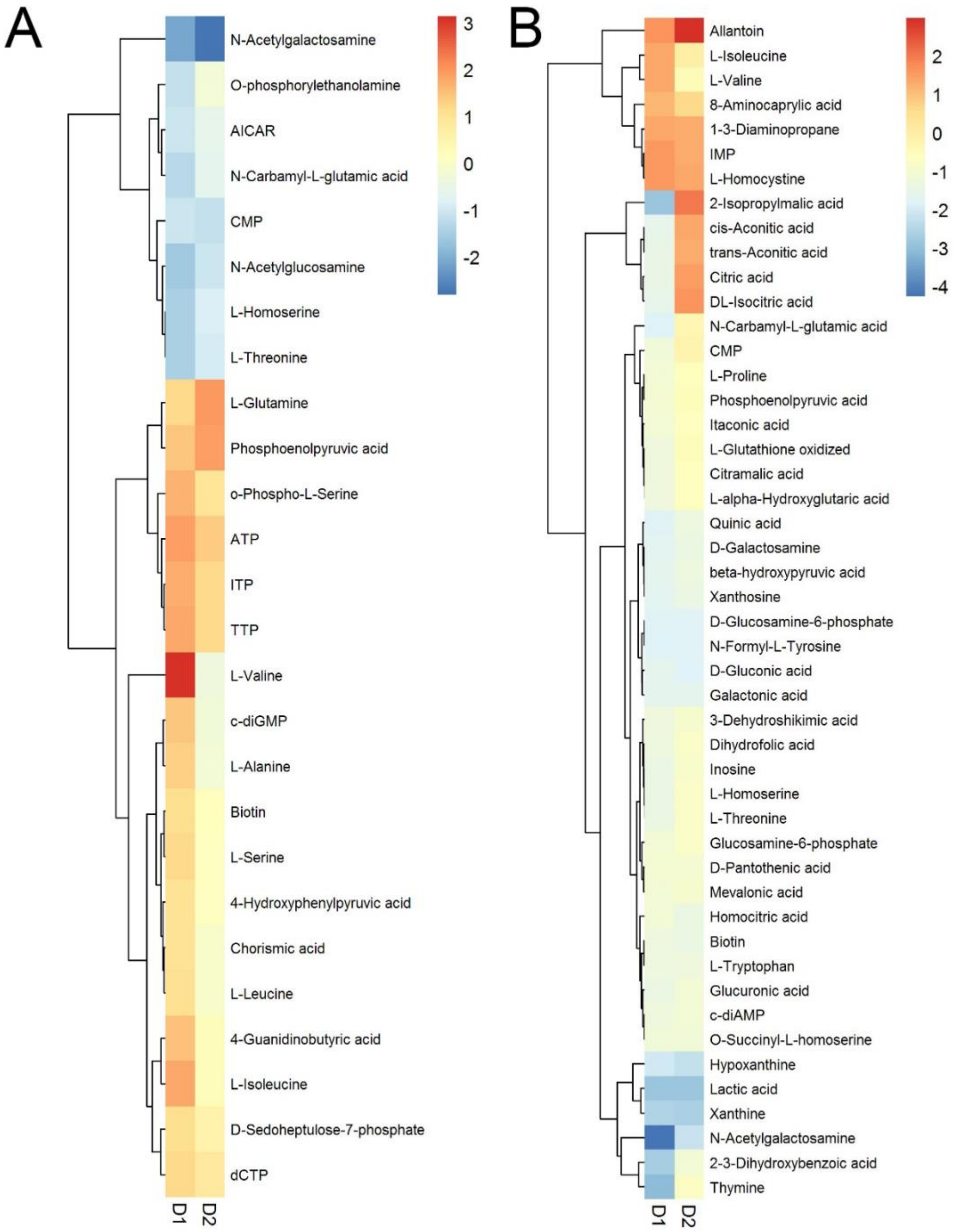

Figure S5. Metabolomics analysis underscores the pattern of shifting metabolism reflected by transcriptional analysis.

The same data used for Figure 7 is presented here with Δ*codY* as the D1 and Cm-treated as the D2 for comparison. Metabolic profiles of Δ*codY* (D1) strain at 6 h (**A**) and 24 h (**B**). Metabolites that change ≥1.5-fold (6 h) and ≥2-fold (24 h) in Δ*codY* compared with wild-type strain are listed. The profiles of corresponding metabolites in wild-type strain (D2) upon exposure to 1 μM Cm at 6 h and 24 h.

bioRxiv preprint doi: https://doi.org/10.1101/2024.03.20.585991; this version posted March 20, 2024. The copyright holder for this preprint (which was not certified by peer review) is the author/funder, who has granted bioRxiv a license to display the preprint in perpetuity. It is made available under aCC-BY 4.0 International license.

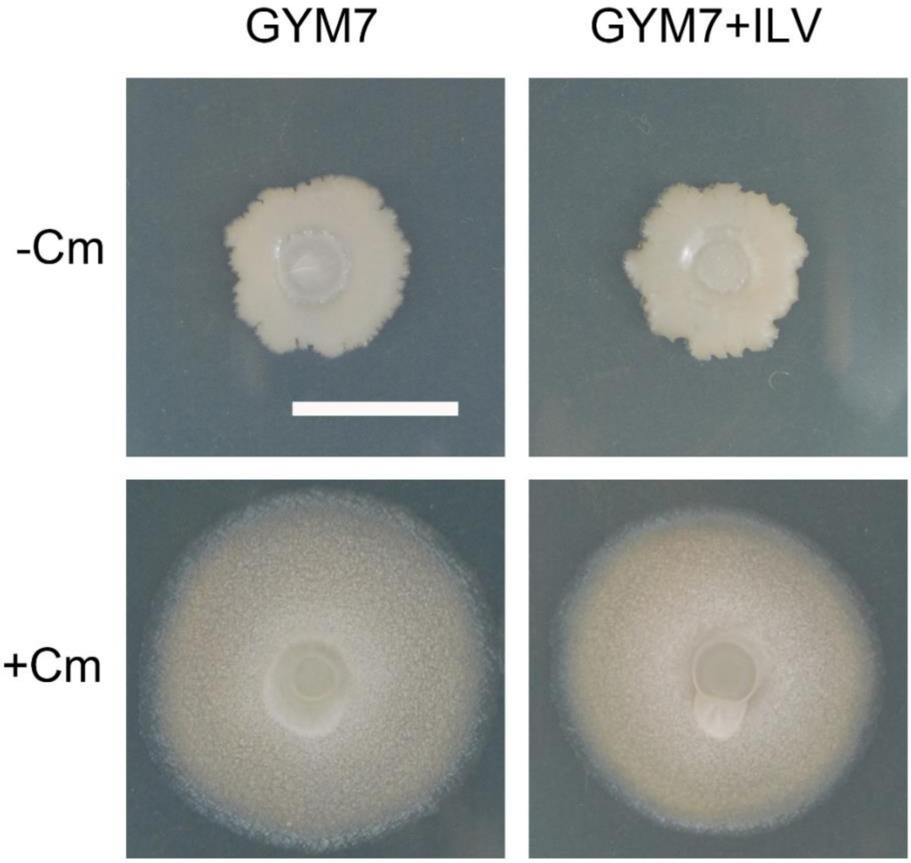

Figure S6. **Supplementation of BCAAs was unable to inhibit sliding motility in the presence of chloramphenicol.**

Wild type B. subtilis NCIB3610 was spotted on the GYM7 plate and GYM7 supplemented with 10 mM each of isoleucine, leucine and valine in the absence (-Cm) and presence (+Cm) of chloramphenicol. Pictures were taken at 24 h. Bar, 1 cm.
